# Surgical Sealing of Laterally Localized Accessory Root Canal with Resin Containing S-PRG Filler in Combination with Non-Surgical Endodontic Treatment: A Case Report

**DOI:** 10.3390/dj8040131

**Published:** 2020-11-20

**Authors:** Shizu Hirata-Tsuchiya, Shigeki Suzuki, Takashi Nakamoto, Naoya Kakimoto, Satoru Yamada, Hideki Shiba

**Affiliations:** 1 Department of Biological Endodontics, Graduate School of Biomedical and Health Sciences, Hiroshima University, Hiroshima 734-8553, Japan; shtsuchiya@hiroshima-u.ac.jp (S.H.-T.); bashihi@hiroshima-u.ac.jp (H.S.); 2 Department of Periodontology and Endodontology, Tohoku University Graduate School of Dentistry, Sendai 980-8575, Japan; satoruy@tohoku.ac.jp; 3 Department of Oral and Maxillofacial Radiology, Graduate School of Biomedical and Health Sciences, Hiroshima University, Hiroshima 734-8553, Japan; tnk@hiroshima-u.ac.jp (T.N.); kakimoto-n@hiroshima-u.ac.jp (N.K.)

**Keywords:** accessory canal, endodontic surgery, maxillary incisor

## Abstract

The spread of root canal infection to surrounding periodontal tissue through accessory root canals reduces the success rate of endodontic treatment. In this case, cone-beam computed tomography revealed a lesion (4 mm from the apex) resulting from an accessory root canal of the maxillary left central incisor. First, non-surgical endodontic treatment was conducted but the sinus tract remained. Surgical preparation of the root cavity was then conducted to remove potentially infected dentin surrounding the accessory root canal. The cavity was filled and the foramen was sealed with resin containing bioactive surface pre-reacted glass (S-PRG) filler. The photopolymerized resin was then contoured and polished. In combination with subsequent supportive non-surgical endodontic treatment, a good clinical outcome with the disappearance of the sinus tract and clinical symptoms such as discomfort and pressure pain and the regeneration of the alveolar bone hanging over the cavity was obtained. In this case, the good clinical outcome may have been due to the dentin-adhesive property and durability of the pre-adhesive system and composite resin. The better biocompatibility of S-PRG fillers presumably facilitated periodontal tissue healing.

## 1. Introduction

Dental pulpitis and subsequent apical periodontitis are mainly caused by the carious and traumatic infection of dental pulp tissue and endodontic treatment is needed to remove the invading oral bacteria. Successful treatment rates were found to be 92%, 74% and 88% for primary endodontic treatment and 86%, 67% and 89% for secondary endodontic treatment in the maxillary central incisor, maxillary secondary incisor and maxillary canine, respectively [1]. The presence of anatomical features such as root construction, root curving, ramifications and lateral root canals, which result in bacterial remnants and their products, is considered to reduce the success rate of endodontic treatment [2,3].

Almost all maxillary central incisors possess a single root with a single canal although there are rare exceptions with two or four root canals [4,5,6]. In contrast to the limited variation in the number of root canals, it is well known that 62.2%, 49.1% and 41.9% of maxillary central incisors, maxillary secondary incisors and maxillary canines, respectively, possess accessory canals including ramifications [7]. Most of the accessory canals in these teeth are localized within 3 mm of the apex; therefore, clinicians usually trim within 3 mm of the root tip in the case of apicoectomy, thereby maintaining an appropriate crown to root ratio. However, 15.9%, 19.7% and 19.4% of maxillary central incisors, maxillary secondary incisors and canines, respectively, possess accessory canals more than 3 mm away from the apex [7]. Therefore, non-surgical endodontic treatments alone may not result in favorable clinical outcomes for maxillary incisors and canines when periodontal infection is caused by pathogens in such accessory canals.

Mineral trioxide aggregate (MTA) is used for perforation repair because of its ability to induce cementum tissue formation, thereby facilitating the formation of a physiological periodontal ligament-cementum structure [8]. However, because MTA does not possess a dentin-adhesive property, the cavity for MTA requires at least 3−4 mm thickness to prevent microleakage [9,10]. Moreover, ethylenediaminetetraacetic acid (EDTA) is known to interfere with MTA hydration and consequently decreases the hardness and biocompatibility [11]. To the best of our knowledge, no clinical study has been conducted to evaluate the durability of MTA and the adhesive strength between MTA and dentin against the mechanical and chemical stress of endodontic treatment.

Surface pre-reacted glass (S-PRG) fillers whose outer layer consists of glass ionomer cement secretes fluoride ions, sodium ions, borate ions, aluminum ions, silicate ions and strontium ions [12,13,14,15,16,17,18]. A prototype of S-PRG paste was able to induce mineral formation in vitro [12] and exhibited antioxidant [13] and antimicrobial [14,15] abilities. Furthermore, when compared with non-treated controls, the application of the prototype of S-PRG paste as an intracanal medication in a rat induced apical periodontitis model promoted periapical tissue healing equivalent to that promoted using Ca(OH)2 [16]. The higher ion releasing capacity of S-PRG contributes not only to the antimicrobial ability [14,15] but also to the cellular migrative properties of human gingival fibroblasts through activating an extracellular signal-regulated kinase 1/2 (ERK1/2) [17]. Therefore, recent accumulating evidence indicates the usefulness of S-PRG filler for surgical root sealing of the foramina of accessory root canals and perforation spots although the prototype of S-PRG paste lacked durability and a dentin-adhesive property. Resin containing S-PRG filler is widely used as restorative material of the crown partly due to its durability, dentin-adhesive property, antimicrobial ability and biocompatibility. However, its ability to fill the cavity adjacent to the area where periodontal tissue regeneration is expected such as the perforation spot and trimmed root surface in apicoectomy has not yet been elucidated.

Here, we report a case in which non-surgical endodontic treatment followed by the surgical disinfection of accessory root canals and surrounding infected dentin by cavity preparation and by filling the cavity with resin containing S-PRG filler was performed for a lesion restricted in the middle part of the maxillary incisor. This suggested the importance of the decision to conduct a surgical procedure for endodontic treatment to fill the prepared accessory root with durability and biocompatibility to induce periodontal tissue healing and regeneration.

## 2. Case Report

**Figure 1:**
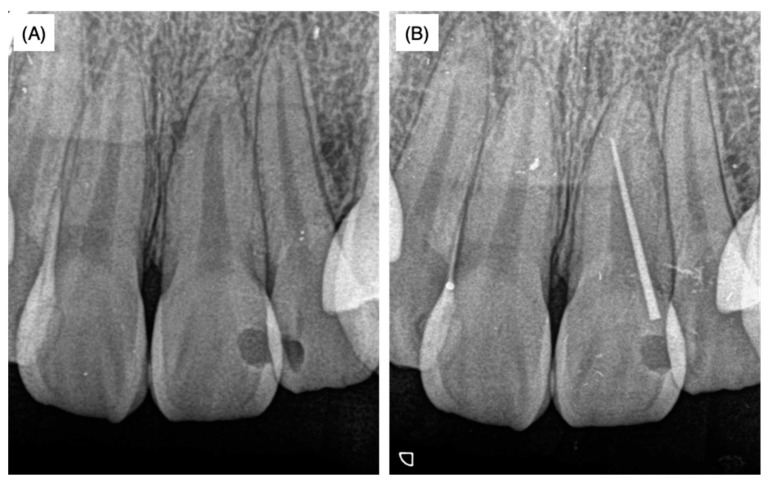
X-ray films without (**A**) and with (**B**) the insertion of gutta-percha points when the patient was 18 years and 8 months old. These X-rays were taken at a private dental clinic before the patient came to our university dental hospital.

A female patient aged 18 years was referred to a private dental clinic for cosmetic problems caused by a black-colored left maxillary central incisor. As swelling in the attached gingiva surrounding the upper left maxillary incisor was observed, X-ray analysis was performed with and without the insertion of gutta-percha points (GC Dental Industrial Corp, Tokyo, Japan) in the sinus tract in order to identify the lesion area (Figure 1A,B).

As there was no response to the electric pulp test in the affected tooth, the pulpal cavity was accessed and standard endodontic treatments including reaming, filing, root irrigation and application of a Ca(OH)_2_ water-based paste (Calcipex II^®^, Nippon Shika Yakuhin Co., Ltd., Yamaguchi, Japan) were conducted three times. The patient was then introduced to Hiroshima University Hospital and the above details and dental radiographs (Figure 1A,B) were provided in the response letter from the dentist at the private clinic to our inquiry. According to the history-taking at the university hospital, when the patient was around 10 years old, the tooth developed severe caries and was restored using composite resin at another private dental clinic. As the patient did not remember the name and address of the dental clinic where this initial treatment was performed, no further information on the initial treatment was available.

**Figure 2:**
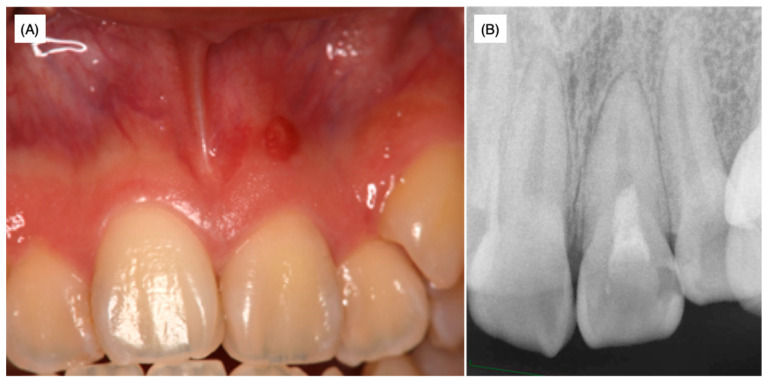
Preoperative intraoral photograph (**A**) and dental radiophotograph (**B**).

The medical histories of the patient and her family were non-contributory and no traumatic history of the frontal teeth was revealed by history-taking. At the time of the first examination at the university hospital, swelling was observed and the left maxillary incisor was discolored (Figure 2A).

Slight pressure pain and notable fistulization were identified in the swollen area. However, no spontaneous pain or significant perpendicular or horizontal percussion pain was identified. The probing depth of the left maxillary incisor was within 2 mm and tooth movement was within physiological limits; thus, root fracture was not assumed. The palatal side was sealed with hydraulic cement. Dental radiographs taken at the first visit to the university hospital as well as radiographs provided by the private dental clinic revealed that the width of the root canal adjacent to the apex of the left maxillary incisor was narrower than that of the maxillary right central incisor (Figure 1 and Figure 2B). The upper two-thirds of the crown side were filled with Calcipex II^®^ from the previous treatment. Moreover, the width of the periodontal ligament space was slightly enlarged (Figure 1 and Figure 2B).

The maxillary left central incisor was isolated with a rubber dam. Standard endodontic treatments including the measurement of the root canal length by electronic apex locator, reaming, filing and root irrigation were conducted. An electron apex locator indicated an orifice of the lateral accessory canal at the middle of the root because the working length at which the meter indicated the apex was shorter than expected. A slight exudate infiltrated on the paper point when it reached the orifice of the lateral accessory canal through the main root canal. The patient felt pain when endodontic files were inserted over the orifice of the lateral accessory canal.

**Figure 3:**
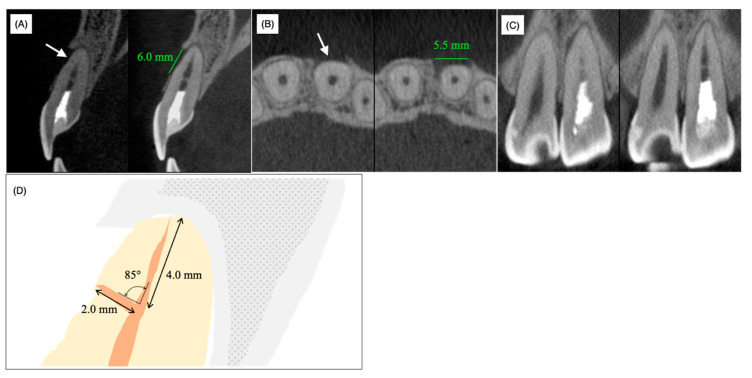
Preoperative cone-beam computed tomography images (**A**) sagittal, (**B**) horizontal and (**C**) coronal. The arrow in (**A**) indicates the lateral foramen. (**D**) Schematic view of the sagittal section of the upper left incisor.

Cone-beam computed tomography (CBCT) demonstrated a horizontal accessory canal at the labial side of the root in the sagittal section (Figure 3A, arrow), bone resorption (length = 6 mm, width = 5.5 mm) whose center was positioned at the foramen of the horizontal accessory canal on the horizontal section (Figure 3B, arrow) and local enlargement of the periodontal ligament space on the mesial side of the middle part of the root (Figure 3C). In contrast to the labial and medial sides of the middle part of the root, the bone surrounding the apical apex was not resorbed and labial bone resorption did not reach the apical apex (Figure 3A,C). A schematic view of the periapical region based on CBCT analyses showed 4.0 mm and 2.0 mm distances from the anatomical apex to the orifice of the laterally localized accessory root canal and from the foramen to the orifice of the laterally localized accessory root canal, respectively; the angle between the main root canal and the laterally localized accessory root canal was 85°, which verified that the laterally localized accessory root canal occurred naturally rather than being caused by an iatrogenic perforation (Figure 3D).

The above findings suggested that inflammation spread into the periodontal ligament tissue from the horizontal accessory canal but not from the apical apex and this case was diagnosed as laterally localized inflammation caused by infection of the accessory root canal. Therefore, due to continuous exudate from the accessory root canal, we considered it prudent to discontinue the non-surgical endodontic treatment and proposed exploratory surgery to the patient in order to directly examine and disinfect the lateral accessory canal under a microscope. We obtained informed consent from her and her mother.

**Figure 4:**
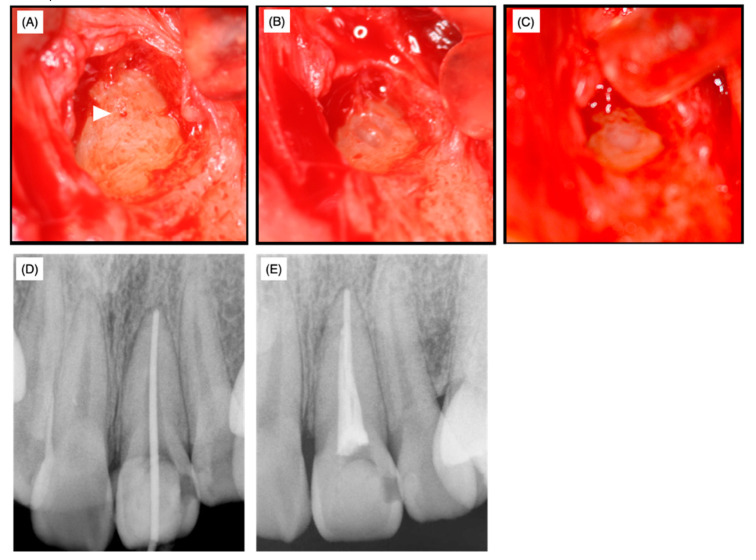
Operative photographs after granular tissue removal (**A**), after cavity preparation (**B**) and after sealing with composite resin (**C**). The arrowhead in (**A**) indicates the lateral foramen. X-ray films before (**D**) and after (**E**) root filling. The fitness of gutta-percha points in the apex was examined (**D**).

At the time of the surgery, local anesthesia was administered, the target side was opened and granulation tissue was removed. The foramen of the accessory canal was identified by microscopy (Figure 4A, arrowhead) and the dentin tissue surrounding the foramen was drilled out using a diamond bur of 0.5 mm in diameter to remove potentially infected surrounding dentin (Figure 4B). After the cavity was disinfected with 10% sodium hypochlorite (NeoCleaner^®^, Neo Seiyaku, Nagoya, Japan) and washed with saline, a No. 25 K-file was inserted into the main canal in order to prevent resin material from leaking into the main canal, thereby preventing subsequent non-surgical endodontic treatment. Subsequently, the cavity was treated using a self-etching dentin-adhesive system (Clearfil MegaBond^®^, Kuraray Medical, Okayama, Japan) and filled with resin (Beautifil flow F02^®^, Shofu, Kyoto, Japan) (Figure 4C). Twelve days after the surgery, no swelling or clinical symptoms were observed, the sutures were removed and the affected area was irrigated with saline.

Over the next 40 days, supportive non-surgical endodontic treatment was conducted five times. At this stage, the root canal length was successfully measured and a working length of 25.0 mm from the top of the crown was set. The apex was enlarged until No. 55 with a K-file and the prepared root canal was irrigated with 3% EDTA solution (Smearclean^®^, Nippon Shika Yakuhin Co., Ltd., Yamaguchi, Japan). Calcipex II^®^ was used as a drug for the root canal and glass polyalkenoate cement (Base Cement^®^, Shofu, Kyoto, Japan) was used as a temporary sealing. The fitness of the gutta-percha points (GC Dental Industrial Corp, Tokyo, Japan) in the apex region was examined (Figure 4D) and then the root was filled by the lateral condensation method using Grossman’s root canal sealer (Nishika Canal Sealer Eugenol Quick E-Q^®^, Nippon Shika Yakuhin Co., Ltd., Yamaguchi, Japan) (Figure 4E).

**Figure 5:**
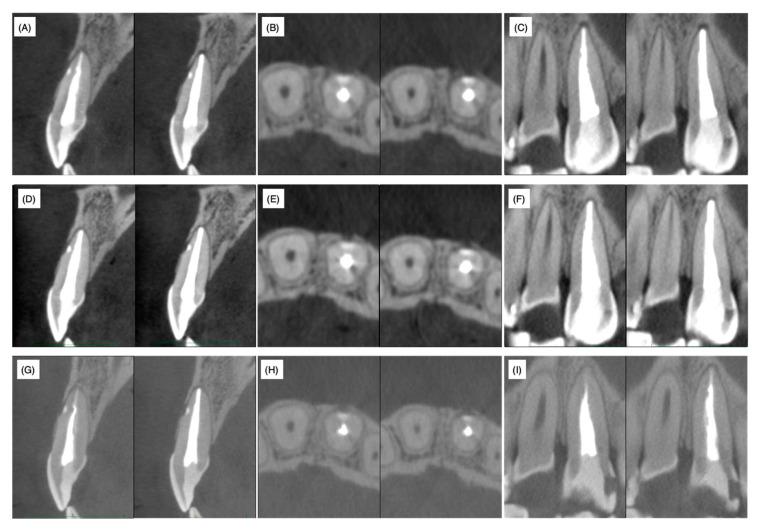
Cone-beam computed tomography images at three months (**A**) sagittal, (**B**) horizontal and (**C**) coronal; one year (**D**) sagittal, (**E**) horizontal and (**F**) coronal and two years (**G**) sagittal, (**H**) horizontal and (**I**) coronal after root filling.

Fourteen days after root canal obturation, the accessible palatal cavity was filled with the composite resin for a permanent restoration. Three months, one year and two years after root canal obturation, CBCT analyses were performed. The lateral canal remained closed by the filled resin material even after two years and the absence of labial bone resorption was chronologically confirmed on cross, sagittal and horizontal sections (Figure 5). Furthermore, no recurrence of gingival swelling was confirmed on photographs and no clinical symptoms were observed.

## 3. Discussion

As described above, 62.2% of maxillary central incisors have access to periodontal ligament tissue other than the apex foramen [1]. However, apical periodontitis mostly spreads from the apex, presumably because the diameter of the apex foramen is larger than that of the lateral foramen and ramifications [19,20]. Moreover, pulp tissue in accessory canals may survive and be immunologically resistant to bacterial invasion from the main root canal even when apical periodontitis with a radiolucent area is noted [20]. The average diameter of the main apical foramen in the maxillary frontal teeth is 0.28 mm according to Chapman et al. [21] and 0.31 mm according to Abdullah et al. [22]. Indeed, the apical foramen was narrow (Figure 1 and Figure 2) and a thin dentin bridge-like radiopaque white line was identified at the horizontal level of the lateral root foramen by CBCT analysis (Figure 3). Thus, we hypothesized that the pulp tissue in the apical region had calcification ability. As prolonged pulp inflammation is known to be related to pulp calcification such as the pulp stone [23] we assumed that the bacterial remnants from the primary carious treatment received at around 10 years of age induced chronic pulpal inflammation, which may have caused the calcification in the apical region. As we were unable to feel any dentin bridge-like hard tissue by hand during non-surgical endodontic treatment, this hard tissue was suggested to be fragile and flimsy.

The successful prognosis rates of apicoectomy by traditional surgery and microsurgery are 59% and 94%, respectively [24]. The main advantages of the microscopic view during the apicoectomy procedure are that it is easy to identify non-treated root canals and microcracks on the root surface as well as the cut surface. After trimming the root tip, clinicians tend to concentrate on the cut surface; however, clinicians should also explore the remaining foramens of lateral accessory root canals even on the palatal/lingual side. From the point of view of the crown/root ratio influence on the tooth lifespan, the remaining foramen should be sealed rather than undergoing further trimming of the root tip.

Non-surgical treatment was conducted to mechanically and chemically disinfect the main root canal. However, the clinical symptoms and signs did not improve necessitating the need for surgical treatment. A slight exudate infiltrated the paper point only when it reached the orifice of the lateral accessory canal through the main root canal. Therefore, the root canal infection was mainly due to communication with the periodontal area surrounding the foramen of the laterally localized accessory root canal and infection of the accessory root canal and the surrounding dentin. It is widely accepted that surgical treatment should be conducted only when non-surgical endodontic treatment has failed. In the present case, our decision for surgical treatment might be considered to have been early. We were unable to wait for a tissue healing response under non-surgical treatment due to the limited period of hospital attendance. MTA, composite resin, glass polyalkenoate cement and o-ethoxybenzoic acid cement (EBA cement) are the alternative choices for filling materials of perforation spots. Among these materials, MTA is widely accepted as the best from a periodontal tissue regeneration point of view. However, MTA requires 3−4 mm of thickness to block microleakage [9,10]. In the present case, the horizontal accessory canal had a length of 2 mm at the labial side of the root in the sagittal section (Figure 3); therefore, in order to prevent detachment of the filling material during subsequent supportive mechanical and chemical endodontic treatments, the self-etching dentin-adhesive system and composite resin were selected based on their strong adhesive strength and chemical stability. The composite resin (Beautifil Flow F02^®^, Shofu) used in this case contains an S-PRG filler and is categorized as a giomer [25]. When the coronally positioned flap operation was conducted to restore gingival recession, the gingival epithelium was re-attached to the GIOMER surface to construct a long junctional epithelium and the root coverage rate of the GIOMER surface was higher than that of glass ionomer cement and GIOMER-free composite resin surfaces [26]. Thus, the relatively higher biocompatibility of the GIOMER may have reduced the radiolucent area and regenerated alveolar bone tissue even in the area hanging over the resin at one and two years after the surgery (Figure 5D–I). MTA has been applied to seal perforations in roots and the bottom of the pulp chamber with a good prognosis, without inducing periodontal tissue inflammation in clinical settings and in in vivo experimental animal models [27,28]. According to a meta-analysis of the treatment outcome of repaired root perforations [29], an overall success rate of 72.5% was estimated for non-surgical repair of root perforations and the rate increased to 80.9% only when MTA application cases were included. In a long-term evaluation of the repair of root perforations with MTA application [30], which was one of the clinical analyses used in the reported meta-analysis [29], teeth were classified into a ‘healing’ group if only two or less of the five clinical and radiographic findings, namely, radiolucency adjacent to the perforation site, continuing root resorption, clinical signs, clinical symptoms and loss of function, were identified at the time of evaluation. If we apply this rule in the present case, radiolucency was adjacent to the perforation site, clinical signs (the presence of sinus tract) and clinical symptoms (discomfort and pressure pain) were identified and only radiolucency adjacent to the perforation site was identified at pre- and postoperative stages (both post one and two years). These scoring results implied that the lesion was healed. Therefore, by considering the reduced radiolucent area at post one and two years, the resin containing S-PRG filler used in the present case can be regarded to possess an apparent regenerative ability equivalent to MTA cements. The possibility of minor leakage between dentin and MTA cement or MTA dropout due to mechanical and chemical stress was not excluded before treatment. Therefore, in addition to the application of MTA only being authorized for pulp capping treatment (not for perforation repair) under the Japanese health insurance system, we selected resin containing S-PRG filler as the filling material in this case.

## 4. Conclusions

This case report strongly recommends dentists to consider the existence of accessory root canals while conducting endodontic treatment of maxillary incisors. As there are several advantages to performing surgical endodontic treatment for maxillary incisors including easy access to the lesion area with minimum bone fenestration due to the thin labial cortical bone and ease in securing the operative field, surgery should be considered if the primary non-surgical treatment fails to cure the laterally localized accessory root canal. Resin containing S-PRG filler should be an ideal sealing material for the cases without securing sufficient material thickness, considering its regenerative capability, adhesive properties and durability.
